# A novel autosomal recessive TERT T1129P mutation in a dyskeratosis congenita family leads to cellular senescence and loss of CD34+ hematopoietic stem cells not reversible by mTOR-inhibition

**DOI:** 10.18632/aging.100835

**Published:** 2015-11-06

**Authors:** Clemens Stockklausner, Simon Raffel, Julia Klermund, Obul Reddy Bandapalli, Fabian Beier, Tim H. Brümmendorf, Friederike Bürger, Sven W. Sauer, Georg F. Hoffmann, Holger Lorenz, Laura Tagliaferri, Daniel Nowak, Wolf-Karsten Hofmann, Rebecca Buergermeister, Carolin Kerber, Tobias Rausch, Jan O. Korbel, Brian Luke, Andreas Trumpp, Andreas E. Kulozik

**Affiliations:** ^1^ Department of Pediatric Oncology, Hematology and Immunology, University of Heidelberg and Molecular Medicine Partnership Unit, 69120 Heidelberg, Germany; ^2^ Division of Stem Cells and Cancer, German Cancer Research Center (DKFZ), Im Neuenheimer Feld 280, 69120 Heidelberg, Germany; ^3^ Heidelberg Institute for Stem Cell Technology and Experimental Medicine (HI-STEM gGmbH), Im Neuenheimer Feld 280, 69120 Heidelberg, Germany; ^4^ German Cancer Consortium (DKTK), 69120 Heidelberg, Germany; ^5^ Zentrum für Molekulare Biologie der Universität Heidelberg (ZMBH), DKFZ-ZMBH Alliance, 69120 Heidelberg, Germany; ^6^ Department of Hematology, Oncology, Hemostaseology and Stem Cell Transplantation, Medical Faculty of the RWTH Aachen University, 52062 Aachen, Germany; ^7^ Center for Pediatric and Adolescent Medicine, Heidelberg University Hospital, Im Neuenheimer Feld 430, 69120 Heidelberg, Germany; ^8^ Department of Hematology and Oncology, University Hospital Mannheim, Medical Faculty Mannheim of the University of Heidelberg, 68167 Mannheim, Germany; ^9^ European Molecular Biology Laboratory (EMBL), Genomics Core Facility, D 69117 Heidelberg, Germany; ^10^ European Molecular Biology Laboratory (EMBL), Genome Biology Unit and Molecular Medicine Partnership Unit, D 69117 Heidelberg, Germany; ^11^ Institute of Molecular Biology gGmbH, gefördert durch die Böhringer Ingelheim Stiftung, 55128 Mainz, Germany

**Keywords:** TERT, TERC, mTOR, rapamycin, sirolimus, senescence

## Abstract

The TERT gene encodes for the reverse transcriptase activity of the telomerase complex and mutations in TERT can lead to dysfunctional telomerase activity resulting in diseases such as dyskeratosis congenita (DKC). Here, we describe a novel TERT mutation at position T1129P leading to DKC with progressive bone marrow (BM) failure in homozygous members of a consanguineous family. BM hematopoietic stem cells (HSCs) of an affected family member were 300-fold reduced associated with a significantly impaired colony forming capacity in vitro and impaired repopulation activity in mouse xenografts. Recent data in yeast suggested improved cellular checkpoint controls by mTOR inhibition preventing cells with short telomeres or DNA damage from dividing. To evaluate a potential therapeutic option for the patient, we treated her primary skin fibroblasts and BM HSCs with the mTOR inhibitor rapamycin. This led to prolonged survival and decreased levels of senescence in T1129P mutant fibroblasts. In contrast, the impaired HSC function could not be improved by mTOR inhibition, as colony forming capacity and multilineage engraftment potential in xenotransplanted mice remained severely impaired. Thus, rapamycin treatment did not rescue the compromised stem cell function of TERT^T1129P^ mutant patient HSCs and outlines limitations of a potential DKC therapy based on rapamycin.

## INTRODUCTION

Telomeres, the protective nucleoprotein structures at chromosome ends, shorten upon each cell division due to the so-called “end-replication problem” [1, 2]. The end-replication problem is compensated for by the reverse transcriptase, telomerase, which is active in germ cells, cancer cells and, to an extent in somatic stem cells [3]. Accelerated telomere shortening leads to the premature replicative senescence of cells and can be caused by mutations of the telomerase components DKC1 (dyskerin), TERC and TERT, among other genes involved in telomere maintenance [4–7]. TERC and TERT represent the RNA and catalytic protein moieties of the telomerase reverse transcriptase, respectively. Mutations affecting the function of these genes may lead to dyskeratosis congenita (DKC), a disease with a highly heterogeneous phenotype [8–11]. Affected patients suffer from a variable combination of skin, nail and mucosal dystrophies, but also life-threatening conditions such as progressive bone marrow failure, pulmonary fibrosis and an increased propensity to develop malignant tumors [12–16]. Telomere loss has been proposed to eliminate cells with a long proliferative history, and in this manner, acts as a tumor suppressor to limit replicative capacity. Telomere attrition also occurs with age and the associated accumulation of senescent cells may contribute to the aging process [13]. In disease states with reduced stem cell replicative reserve, substantially increased stem cell turnover or in the absence of telomerase activity short telomeres accumulate in hematopoietic stem cells [17]. Critically short telomeres are dysfunctional in terms of chromosome end protection and hence upon nucleolytic processing the DNA damage checkpoint is unleashed, thereby driving the onset of replicative senescence [18]. Dysfunctional telomeres are also prone to unscheduled repair events leading to chromosomal rearrangements. Therefore, in the absence of a functional DNA damage checkpoint, chronic telomere shortening could also potentially lead to pathogenic chromosomal instability.

Current treatment for patients affected by dyskeratosis congenita includes the androgen danazol [19–21]. The use of androgens can lead to virilization in female patients and thereby limits its therapeutic range [22, 23].

Stem cell transplantation to cure the progressive bone marrow failure is challenging, and DKC patients have a poor tolerance for conditioning regiments and frequently suffer from life threatening side effects [24–26]. Future therapy options include the utilization of induced pluripotent stem cells that might be beneficial for patients that have defined mutations in telomerase components such as TERC [5].

mTOR is a protein kinase that promotes cell growth in response to nutrient supplies and growth signals, and can be specifically inhibited by rapamycin [27]. As it has been shown that inhibiting the mTOR pathway with rapamycin reduces the rate of cellular senescence onset, we hypothesized that rapamycin might have a therapeutic potential for patients suffering from mutations of the telomerase complex where senescent cells accumulate [28, 29].

In this work we describe a consanguineous Libyan family in which we identify a novel T1129P TERT mutation leading to progressive bone marrow failure in homozygous family members. In order to test our hypothesis that rapamycin may rescue or at least improve the physiology of TERT^T1129P^ patient cells, we analyzed the effect of the mTOR inhibitor rapamycin on growth and senescence of skin fibroblasts and on hematopoietic stem cells using in vitro cultures and xenograft mouse models.

## RESULTS

### The novel TERT T1129P mutation leads to pathological telomere shortening causing progressive bone marrow failure in homozygous patients

**Figure 1:**
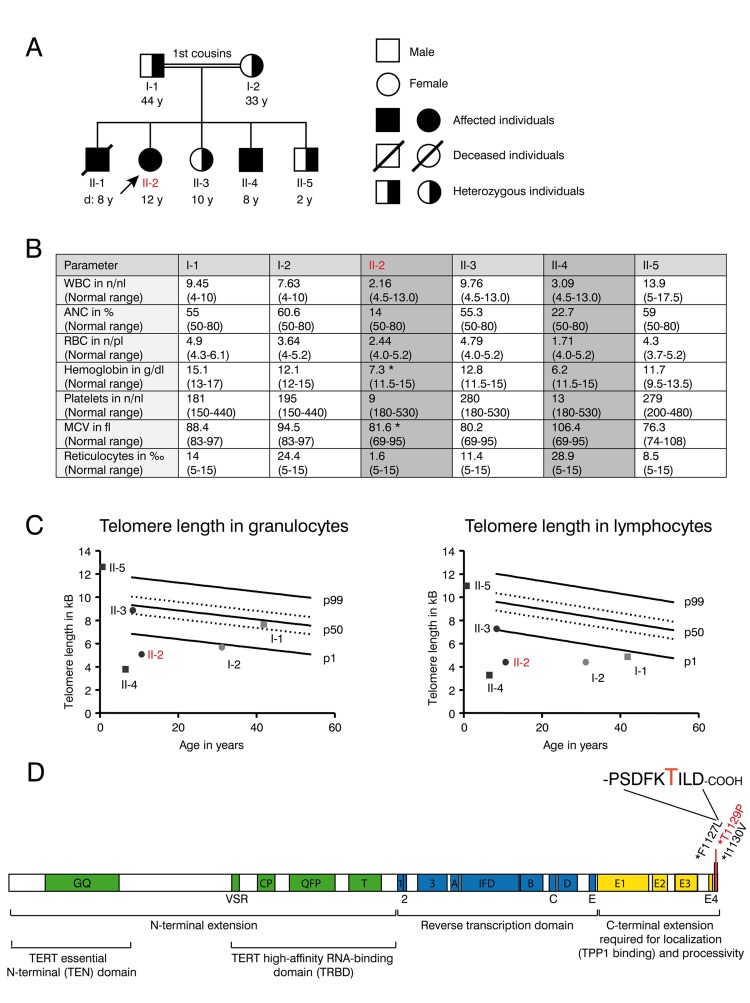
Clinical features and telomere length of the DKC family with the novel T1129P TERT mutation (**A**) Family tree of the consanguineous Libyan family. Family members affected by dyskeratosis congenita (son II-1 (deceased), daughter II-2, son II-4) are indicated in black. Marked in red: patient II-2 who was analyzed in detail in the following figures. (**B**) Table showing complete blood counts (WBC=white blood cells (n/nl), ANC= absolute neutrophil count (% of WBC), RBC= red blood cells (n/pl), Hb=hemoglobin (g/dl), Plt=platelet count, mean corpuscular volume (MCV in fl) and reticulocytes (**2030** of RBC) and the respective age dependent normal values in brackets of the family members I-1, I-2, II-2, II-3, II-4 and II-5 shown in (A). Family members II-2 and II-4 that were diagnosed with dyskeratosis congenita and were homozygous for the TERT^T1129P^ mutation are highlighted with grey color. Indicated with *: Patient II-2 was on a 3-weekly red cell transfusion regimen and had a red cell transfusion of 15 ml/kg erythrocytes 20 days before the sample was taken; patient II-4 had no history of red blood cell or platelet transfusions. (**C**) Telomere lengths of the described family determined in lymphocytes and granulocytes of the peripheral blood. Absolute telomere lengths in kb of lymphocytes and granulocytes of the patient II-2, her affected brother II-4, her siblings II-3, II-5 and her parents I-1 and I-2 are shown in the context of age-dependent percentiles (Females: circle, males: square. Parents: light grey, children: black. Marked in red: patient II-2 who was analyzed in detail). The solid lines represent the respective 1%, 50%, 99% percentile curves. The dashed lines represent the 25% and 75% percentile. (**D**) Schematic representation of the TERT gene with functional domains and known mutations at the C-terminus. Our novel T1129P mutation is depicted in red.

Progressive bone marrow failure including transfusion dependent anemia und thrombocytopenia was first diagnosed in patient II-1 at the age of six years in a consanguineous Libyan family when a blood count was obtained to address symptoms of anemia including weakness and pallor. There was no history of transfusions in the family before. The parents of II-1 were first degree cousins and family studies showed similar thrombocytopenia and anemia in two of their other offspring (II-2 and II-4) (Figure 1 A and B). Normal white blood counts, hemoglobin and platelet counts were observed from the father (I-1), mother (I-2) as well as in the second sister (II-3) and in the third brother (II-5). No family member showed any indication of nail dystrophy or skin alterations.

II-1 died at the age of eight years during conditioning regimen for intended stem cell transplantation that was performed abroad under the suspected diagnosis of aplastic anemia. His aplastic anemia was not diagnosed on a molecular basis and blood samples are no longer available.

The patient II-2 presented at our center at the age of 12 years with progressive bone marrow failure including transfusion dependent anemia and thrombocytopenia, leukopenia (Figure 1B) and hypermenorrhea. Fanconi anemia was excluded as normal results were obtained for the analysis of DNA breakage. Suspected dyskeratosis congenita was confirmed by telomere length analysis in lymphocytes and granulocytes as determined by Flow-FISH (Figure 1C). The telomere lengths in lymphocytes and granulocytes in patients II-2 and II-4 and the mother I-2 corresponded to less than the 1^st^ percentile of age matched controls. Telomere length of the father (I-1) in lymphocytes (right panel) also corresponded to less than the 1^st^ percentile of age-matched controls, whereas it was normal in granulocytes (left panel). The healthy sibling II-3 also displayed a decreased telomere length when compared to age matched controls, although not below the 1^st^ percentile (Figure 1C). Pulmonary function was normal in patient II-2 and not determined in other family members.

Whole exome sequencing and validation by Sanger sequencing revealed a novel homozygous c. 3385 A>C mutation (nucleotide entry NM_198253), resulting in the novel p.Thr1129Pro or T1129P mutation (Figure 1D) of the TERT gene in the 12-year-old patient II-2. No other homozygous mutation was found in the patient. This mutation was absent from public SNP databases (dbSNP, 1000 Genome variant catalogue), conserved and was predicted to be damaging using SIFT and PolyPhen-2 [30, 31]. The unaffected family members were heterozygous carriers of this mutation. The 8-year-old brother II-4 was detected with the same homozygous mutation by Sanger sequencing from peripheral blood.

### The T1129P mutated TERT does not show nuclear clustering together with TERC in a ST-cell culture model

**Figure 2:**
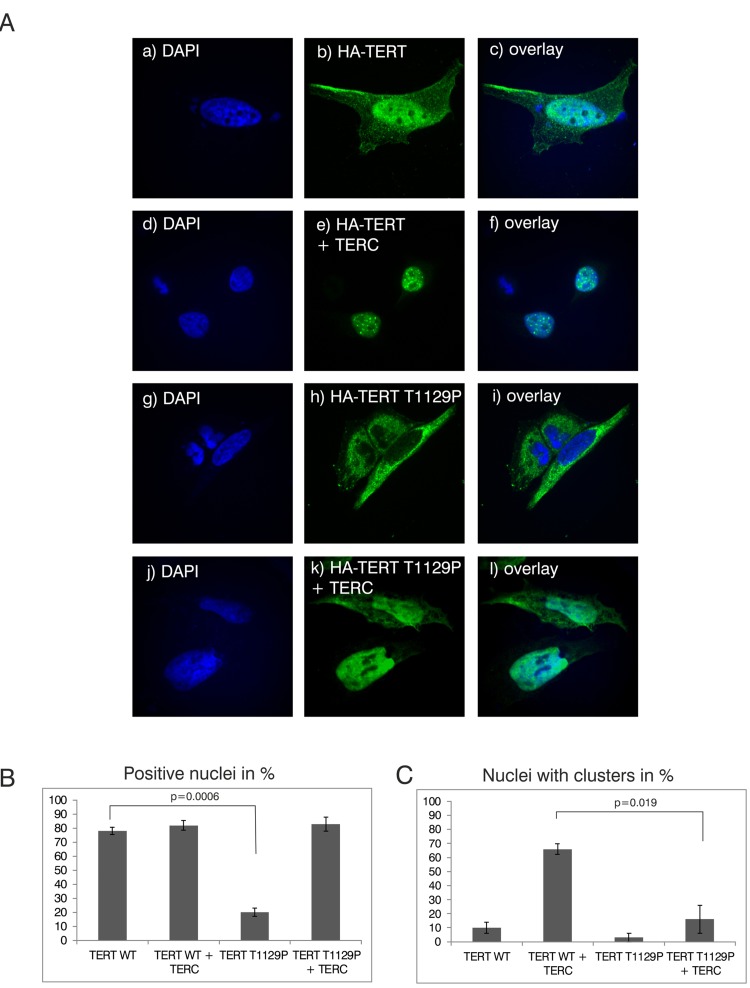
Analysis of nuclear clustering of the TERT T1129P mutation in a cell culture model (**A**) Representative confocal images of transiently co-transfected HeLa-cells. HA-TERT or HA-TERT T1129P, respectively, harboring 3 HA-tag sequences in frame at the N-terminus were transfected and fixed 48h later. On the indicated pictures e) and k) equal amounts of the TERC minigene was co-transfected together with the respective TERT minigene. The HA-tag was visualized with a mouse monoclonal anti-HA antibody and an anti-mouse secondary antibody linked to Alexa 488 as described in Materials and Methods. The nucleus was visualized by DAPI staining. The panels on the right represent overlays of the TERT wild type or the T1129P mutations, respectively, with the nucleus stained with DAPI. Cells depicted represent the subcellular distribution pattern seen in >90% of the transfected cells. (**B**) and (**C**) Quantification of nuclear accumulation and clustering. For quantification of nuclear accumulation and clustering in the nuclei, 100 cells each from 3 independent transfections have been assessed and counted visually for the presence of nuclear staining and/or nuclear clustering. For statistical analysis a student's unpaired two-tailed t-test was used.

The novel T1129P mutation is located at the C-terminus of TERT altering the 4^th^ last amino acid (Figure 1D). This region of normal TERT was shown to bind the telomeric protein TPP1, and has therefore been suggested to be required for the telomeric localization of TERT [32]. To investigate if this novel mutation would influence recruitment of the telomerase complex, we employed a modified “supertelomerase” assay (ST) that used transient, plasmid based expression of the central scaffold protein of the telomerase ribonucleo-protein TERC and hemagglutinin (HA)-tagged TERT in HeLa cells [32–35]. As shown in Figure 2, transfection of N-terminally HA-tagged TERT into HeLa cells resulted in a nucleoplasmic pattern. In line with previous findings, expression of TERC together with HA-TERT resulted in nuclear clusters that have been described as clusters with the telomeres at the chromosomal ends (Figure 2A, compare b with e) [32]. Interestingly, the mutant HA-TERT T1129P protein was detected in the cytoplasm in the absence of co-expressed TERC. When co-expressed with TERC, a nucleoplasmic pattern was observed, but it lacked the typical nuclear clustering of wild type TERT (Figure 2A, compare e to h, k, quantified in B and C) suggesting a failure to be recruited to chromosome ends. Taken together, these results strongly further support the hypothesis that the T1129P mutation impairs the recruitment of TERT to the telomerase complex located at its site of action at the chromosome ends.

### mTOR inhibition with rapamycin influences population doublings and senescence of patient skin fibroblast cultures

The proliferative capacity of cells can by quantified by calculating the population doublings that cells undergo during the culturing process as a function of time. Population doublings are defined as the number of times that the cell number is doubled. As telomeres shorten, the proliferative potential decreases. Generally, population doublings can be recorded in skin fibroblast cultures by plating a specific number of cells and then counting those cells after a defined period of growth as described in Material and Methods. Skin fibroblasts represent an easily accessible cell type that can be cultivated over a long time period, and were treated with either 5 nM rapamycin or DMSO as a vehicle control. Fibroblast cultures obtained from the father (I-1) fulfilled criteria of senescence from the beginning, as they did not show population doublings within 4 weeks despite frequent changes of cell culture media (not shown).

**Figure 3:**
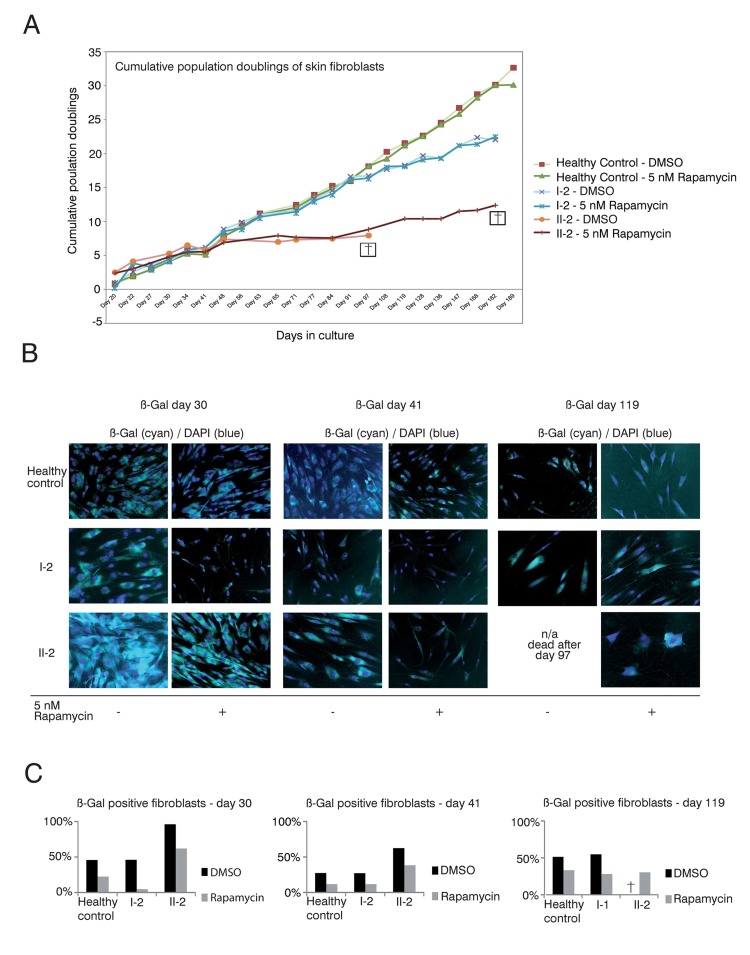
Rapamycin treatment of DKC skin fibroblast cultures (**A**) Cumulative population doublings of skin fibroblasts. Fibroblast cultures from the mother I-2, the daughter II-2 and a healthy control were trypsinized and viable cells determined by trypan blue staining. Viable cells remained unstained. Population doublings were calculated using the following equation: PD=X + log2(Y/I) where: X = initial PD I = cell inoculum (number of cells plated in the flask) Y = final cell yield (number of cells at the end of the growth period). Cell were defined as dead (indicated with a cross) if no remaining viable cells were detected. (**B**) Δ-gal senescence assay of skin fibroblasts. Fibroblasts were cultured in DMEM/10% FCS/1%PenStrep containing either 5nM rapamycin dissolved in DMSO or the equal volume of DMSO as negative control (equivalent to a 1:1,000 dilution). For the indicated timepoints, cells were fixed after 24h with 0.1% glutaraldehyde and stained for Δ-galactosidase (Δ-Gal) at pH 6 as described in Materials and Methods. Nuclei were visualized by staining with DAPI (Sigma-Aldrich), diluted 1:10,000. Coverglasses were embedded in moviol 4-88 (Carl Roth) on slides. Cells were observed at 20-fold magnification and pictures taken at brightfield and fluorescent light with a filter set suitable for DAPI on an Olympus CellR microscope. Depicted overlays of brightfield and fluorescence were merged in ImageJ. Images are representative of the indicated time points. (**C**) Quantification of Δ-gal assay. Δ-Gal-positive cells were detected at the indicated time points using a programmed plugin for the image editing program ImageJ as described in Materials and Methods. The quantified images were representative of the indicated time points. 200 cells counted by DAPI staining were analyzed for each time point and measurement. The fibroblasts were determined as Δ-Gal positive when blue staining in the brightfield reached a defined intensity and surrounding area of the core that was detected in the fluorescence light (Ex 330-385, Em LP420 filter set for DAPI detection).

Skin fibroblast cultures of the TERT T1129P homozygous patient II-2 but also from the heterozygous mother I-2 showed impaired population doublings when compared to fibroblasts from a healthy age matched control (Figure 3A). Treatment of the control and mother (I-2) fibroblasts with rapamycin did not influence the proliferative potential measured by population doublings over time (Figure 3A). The fibroblast culture of the patient II-2 did not show a comparable proliferation potential after day 48 and had no vital cells after day 97. In contrast, the rapamycin treated fibroblast culture of patient II-2 still divided (albeit slowly) after day 97 and showed prolonged survival until day 182 (Figure 3A).

The limited capacity of cells to divide culminates in senescence, a status that is characterized by decreased viability, enlarged cell size, altered pattern of gene expression and expression of pH dependent beta-galactosidase activity [36–38]. Δ-galactosidase activity is present only in senescent cells and is not found in pre-senescent, quiescent or immortal cells [37]. Therefore, we performed Δ-galactosidase assays to test if the prolonged survival in the rapamycin treated fibroblast cultures of the patient (II-2) correlated with decreased senescence (Figure 3B and C). We detected decreased senescence in the control fibroblasts, fibroblasts of the mother I-2 and the affected patient II-2 at day 30 when treated with rapamycin (Figure 3B and C). The same effect was observed at day 41 of this experiment. At day 119 the DMSO treated fibroblast culture of our patient II-2 did not show any vital cells (no viability after day 97). In healthy control fibroblasts and the mother's fibroblasts, the rapamycin treated cultures still showed decreased senescence when compared to the respective DMSO treated cultures (Figure 3B). Taken together, these data reveal a positive correlation between proliferative potential and a decrease of the senescence marker, beta-galactosidase activity, in DC patient cells.

### CD34+ HSPCs are reduced more than 300-fold in patient bone marrow

**Figure 4:**
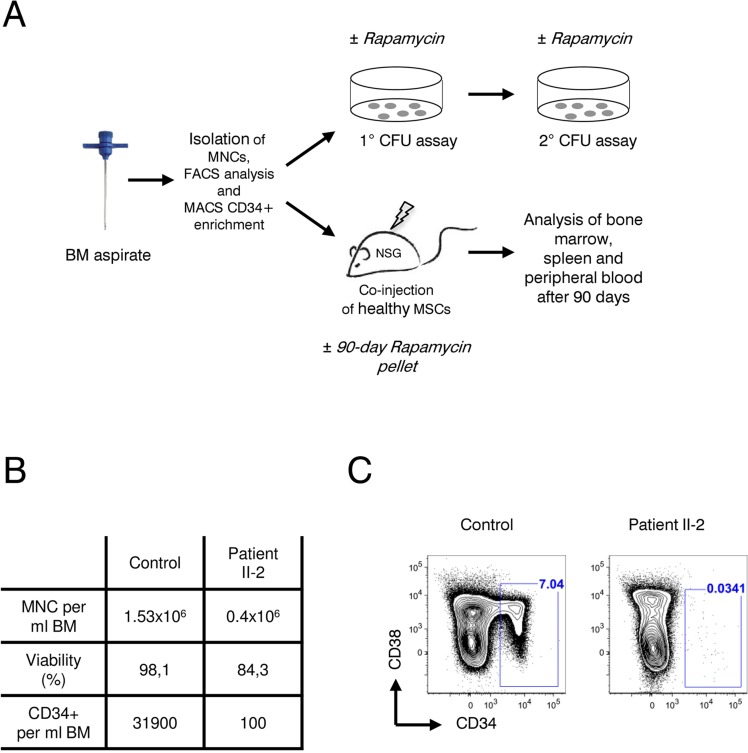
Analysis of patient II-2 bone marrow (**A**) Experimental setup: Bone marrow mononuclear cells were isolated by gradient centrifugation from patient II-2 and healthy control, analyzed by FACS and either plated in colony forming unit assays (see Figure 5) or transplanted into NSG females (see Figure 6). (**B**) Bone marrow characteristics at time of sampling. (**C**) FACS plots showing CD34 and CD38 levels gated on live, lineage-negative cells. Gates show frequencies of CD34+ cells in percent.

Next, we sought to investigate the effect of rapamycin treatment on hematopoietic stem and progenitor cells (HSPCs), hypothesizing that in line with our previous results with fibroblasts (Figure 3), a prolonged survival of HSPCs could improve the patient's blood counts. If so, rapamycin might offer a therapeutic treatment to reduce transfusion dependence of the affected family members. Patient bone marrow derived HSPCs were characterized and quantified by flow cytometry and functionally characterized in vitro by colony forming unit (CFU) assays as well as in vivo by xeno-transplantation into immunocompromized NOD/SCID/interleukin 2 receptor γ^null^ (NSG) mice. Rapamycin treatment and control groups were included in all experiments (Figure 4A). As shown in Figure 4B cellularity and HSPC frequency were strongly reduced in the bone marrow of patient II-2. Compared to a healthy female donor the patient had a 4-fold reduced bone marrow mononuclear cell count (1.53×10^6^/ml vs. 0.4×10^6^/ml) with reduced viability after gradient centrifugation (98.1 percent vs. 84.3 percent viable cells). A dramatic 300-fold reduction was observed in CD34+ HSPCs per ml bone marrow. Only 0.034 percent of all lineage negative cells were CD34+ in the patient II-2 compared to 7.04 percent in the healthy control sample, highlighting a severe HSPC depletion phenotype (Figure 4B and C). Using magnetic bead enrichment for CD34, less than 10,000 CD34+ cells could be isolated from 100 ml bone marrow aspirate, limiting functional studies with these cells.

### Impaired clonogenic growth potential of patient II-2 HSPCs in vitro was not improved by rapamycin

**Figure 5:**
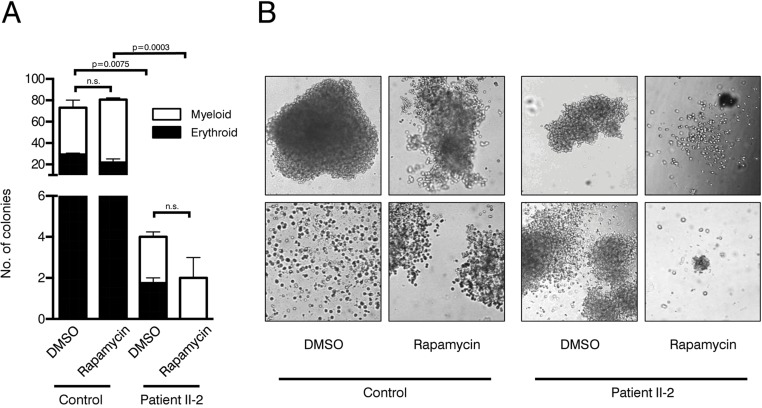
Colony forming unit assays (**A**) Number of colonies in patient and control CFU assays treated with rapamycin or DMSO. Cells were plated in duplicates. Student's t-test was performed on total number of colonies, n.s. not significant. (**B**) Representative pictures of colonies.

To evaluate clonogenicity and lineage differentiation potential of the patient's HSPCs and the influence of rapamycin treatment we performed colony forming unit (CFU) assays (see Figure 4A for experimental design). Plating 3,000 CD34+ cells resulted in approx. 80 colonies per plate in the healthy control. In contrast, the patient's HSPC colony forming potential was significantly reduced, revealing on average only 4 colonies in the DMSO treatment group and 2 colonies in the rapamycin treatment group (Figure 5A). Although colonies treated with rapamycin were reduced in size in both patient and healthy control, this effect was more pronounced in the patient HSPCs (Figure 5B). Furthermore, patient HSPCs showed no long-term self-renewing potential as no colonies were detected anymore in secondary CFU assays, irrespective of rapamycin treatment. In addition to a reduced abundance of HSPCs in the patient's bone marrow, results from the CFU assays indicate a severe functional impairment including self-renewal potential of mutant progenitors. However, in contrast to our observation in fibroblasts, rapamycin treatment showed no beneficial effect on colony number, size and self-renewal activity.

### Xenotransplantation of remaining CD34-negative HSPCs from patient II-2 does not result in multilineage engraftment

**Figure 6:**
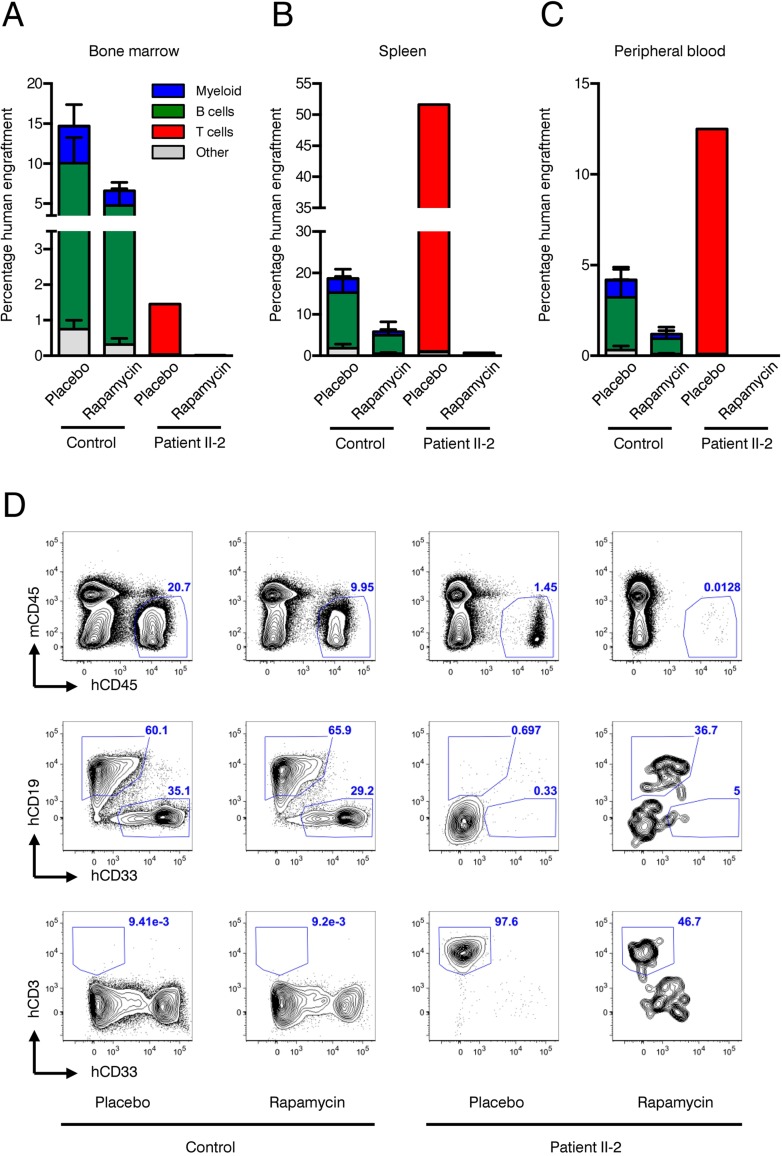
Xenotransplantation experiments (**A**-**C**) Frequencies of human engraftment with respect to lineage differentiation in bone marrow (**A**), spleen (**B**) and peripheral blood (**C**) 90 days after transplantation of patient II-2 or healthy bone marrow. Mice were treated either with rapamycin or placebo. n=2 per condition in healthy donor, n=1 per condition in patient (**D**) Representative FACS plots depicting human (h)CD45 versus mouse (ms)CD45 for blood cell chimerism (upper panel), human (h)CD19 for lymphoid differentiation and human (h)CD33 for myeloid differentiation (middle panel) and human (h)CD3 for T cell expansion. Upper panel is gated on live cells, middle and lower panels are gated on live, hCD45+ cells. Numbers represent percentage of gated events.

Xenotransplantation of human HSPCs into immunocompromized NSG mice is considered the gold standard to evaluate hematopoietic stem cell function. We have recently shown that co-transplantation of mesenchymal stromal cells (MSCs) can enable engraftment of functionally impaired and usually non-transplantable HSPCs derived from Myelodysplastic Syndrome patients [39]. Lacking sufficient amounts of CD34+ HSPCs and to rule out the possibility that the patient's HSPCs are “hidden” within the CD34-negative fraction we co-injected CD34-negative BM MNCs together with healthy human MSCs infra-femorally into sub-lethally irradiated NSG recipient mice. As control, CD34+ healthy HSPCs and MSCs were co-transplanted. In addition, 90-day slow release rapamycin or placebo pellets were implanted subcutaneously. Engraftment of human blood cells was measured by the chimerism for human CD45+ cells 90 days after transplantation. Expression of human CD19 determined lymphoid lineage output, while human CD33 expression was indicative of myeloid differentiation. As expected, healthy HSPCs reconstituted multi-lineage human hematopoiesis in bone marrow (Figure 6A, D), spleen (Figure 6B) and peripheral blood (Figure 6C) of recipient mice. Although not statistically significant, there was a clear trend showing that human CD45+ engraftment was impaired in the rapamycin treatment group in all organs analyzed. In contrast, patient II-2 derived CD34-negative bone marrow MNCs failed to engraft in both rapamycin and placebo treated animals as neither myeloid nor lymphoid cell engraftment was observed.

However, CD3+ T cells, which were present in the transplanted CD34-negative cell fraction of patient II-2, expanded over the course of 90 days under placebo treatment leading to a clinically inapparent graft-versus-host disease. Rapamycin almost completely suppressed T cell expansion in the recipient mice, confirming the activity of the rapamycin pellets and being in line with its known mode of action as an immunosuppressive drug (Figure 6A–D).

Taken together, our experiments with bone marrow MNCs from the dyskeratosis congenita patient II-2 revealed a striking reduction in HSPCs associated with a severe functional impairment of stem cell activity that could not be improved by rapamycin treatment.

## DISCUSSION

Members of the telomerase complex such as TERC, TERT or DKC1 play fundamental roles in aging processes [4–7]. Mutations in these genes may lead to diseases associated with premature aging such as DKC and cancers. Therefore, a refined knowledge of the effects of these mutations may prove useful for understanding pathways that lead to, or mitigate, long-term health, prevention of cancer and late-stage disease. The novel germline T1129P mutation in the TERT gene identified in a consanguineous Libyan family leads to DKC with progressive bone marrow failure in all homo-zygous individuals. Patients II-2 and II-4 showed significantly shortened telomeres below the 1^st^ percentile when compared to healthy controls or to heterozygous family members. The pronounced telomere loss for lymphocytes in comparison to granulocytes is consistent with previous findings in healthy individuals as well as in patients with reduced telomere activity [40, 41]. Only homozygous family members showed progressive bone marrow failure. Heterozygous family members showed normal blood results. Heterozygosity of TERC- and most TERT-mutations can lead to haploinsufficiency and to the clinical phenotype of dyskeratosis congenita, although some TERT mutations cause recessive DKC with heterozygous carriers showing normal blood counts [42]. The heterozygous family members described here are phenotypically healthy despite the presence of shortened telomeres indicating a recessive mode of inheritance of the TERT T1129P mutation. This is consistent with the observation that disease severity cannot be predicted by telomere length alone [43]. However, a late onset of clinical symptoms cannot, of course, be ruled out. Strikingly, the phenotype of affected, homozygous family members only showed progressive bone marrow failure with an absence of other DKC related symptoms such as skin or nail dystrophy or pulmonary fibrosis. The localization of the mutation within the gene does not necessarily predict the phenotype, which is highly variable in various mutations described throughout the TERT gene [4]. Our novel autosomal recessive T1129P mutation is in close proximity to the previously described mutations at positions 1127 and 1130 [43, 44]. When comparing the phenotypes and modes of inheritance of these two near-by mutations, it is striking that the mutation F1127L resembles Hoyeraal-Hreidarsson-syndrome and causes autosomal dominant dyskeratosis congenita whereas the somatic I1130V mutation leads to non-severe aplastic anemia [43, 44].

It still needs to be elucidated how the T1129P TERT mutation exerts its effect causing aplastic anemia in affected patients. In HeLa cell cultures the T1129P mutated TERT did not efficiently enter the nucleus without co-transfected TERC (Figure 2A and B). Together with the lack of nuclear clustering upon co-transfection with TERC, this indicates an inefficient recruitment of the telomerase complex to telomeres within the nucleus for T1129P mutated TERT. As the postulated binding site of TPP1 is within the C-terminus, binding to TPP1 or other members of the telomerase recruitment complex might be impaired by the novel T1129P mutation. A mutant of the three amino acid sequence F1127/K1128/T1129 has been shown before to display only slightly compromised telomerase catalytic activity in vitro, but its in vivo ability to elongate telomeres was highly compromised [45]. In another study, this mutant was further analyzed for its ability to localize to telomeres and to TPP1. While it did not localize to telomeres, it could still localize with TPP1 in Cajal bodies, even though the association with TPP1 was weaker compared to wild type [32].

As androgens harbor various side effects especially for female patients with DKC including virilization, there is need for substances that are safe and well tolerated antagonizing pre-mature aging processes [22, 39]. mTOR inhibition, by rapamycin, has been demonstrated to extend lifespan in a host of model organisms as well as reduce the onset of cellular senescence in cell culture [46, 47]. Moreover, recent data obtained in yeast suggest that mTOR inhibition by rapamycin can strengthen the DNA damage checkpoint and thereby increase the likelihood that cell division only occurs when damaged chromosomes are repaired [48].

Therefore, rapamycin, a substance well characterized in the clinical setting, might be an attractive target to treat DKC patients as they harbor increased numbers of senescent cells which contain dysfunctional chromosome ends. Before administering rapamycin to the patient, we assessed its effect on skin fibroblasts and on HSPCs – the cells that are most severely affected in our patient. Rapamycin had no effect on enhancing the proliferation of normal skin fibroblasts but did prolong the survival of the fibroblasts from the affected patient II-2 (Figure 3A). In our functional analysis, senescence of skin fibroblasts of patient II-2 was increased, as expected. Rapamycin showed an effect on decreasing cellular senescence in treated skin fibroblast cultures of patient II-2. Strikingly, cellular senescence was also reduced in the fibroblast culture of the mother I-2 and the fibroblasts of the healthy control (Figure 3B and C). As mTOR inhibition regulates cellular pathways that affect senescence and aging, this might show the influence of mTOR on fundamental senescence steps even in healthy cells [47]. All fibroblast cultures showed a higher initial senescence at day 30 when compared to day 41. This can be explained by an increased senescence of the newly plated cells and later a reduced senescence of the increasingly dense cell cultures at day 41 that could have been caused by contact inhibition of geroconversion [49, 50].

Decreased frequency of HSPCs and severe impairment of these cells in functional tests such as colony-forming unit assays and xenotransplantation into immuno-compromized NSG mice highlight a fundamental HSC defect in patient II-2 (Figures 4 and 5).

In contrast to fibroblasts, rapamycin treatment did not improve HSC function in these assays. The fact that rapamycin treatment resulted in a trend towards fewer and smaller colonies argues against a possible improvement of the patient's blood counts by inhibiting mTOR. However, we cannot exclude that long-term rapamycin treatment of CD34+ HSPCs may ultimately lead to restoration of HSC function as CFU assays cover only a few weeks. Due to limited availability of diseased CD34+ HSPCs (only 10,000 CD34+ cells in 100ml bone marrow aspirate) xenotransplantation had to be performed using MNCs not selected for CD34. The data thus exclude the possibility that the patient's HSCs in the diseased BM down-regulated CD34 expression. Even co-transplantation of MSCs, which typically enhance engraftment of stem cells usually not capable of initiating human hematopoiesis in NSG mice, showed no beneficial effect [39].

Interestingly, T cells present in the CD34-negative MNCs expanded in the non-treated xenografts, while the immunosuppressive agent rapamycin blocked their expansion efficiently in the treated animals (Figure 6).

Due to the nature of our biological samples (skin biopsy and bone marrow aspirate from patient II-2) repetitive sampling was not possible or useful.

Previous studies in yeast demonstrated that rapamycin was beneficial for cells with dysfunctional telomeres when cells were given the chance to repair the telomeres and eventually proliferate in the absence of rapamycin [48]. Indeed when telomere dysfunction and rapamycin treatment were chronic, there was an initial lag in growth and only at very late time points the rapamycin treatment appeared to be beneficial (J.K. and B.L unpublished results). In the mouse experiments, rapamycin was constantly released from the pellets. In contrast, rapamycin was added to the fibroblast cultures at the time of the splitting procedure and a possible degradation of the drug over time might have limited the toxic effects in our fibroblast experiments. Fine-tuning of rapamycin dosage might therefore be required for the anti-senescent effect and to avoid toxic effects [51]. Therefore it may be necessary to re-evaluate the dosage of rapamycin and the effects of cyclic dosing in order to see stronger effects.

The observed difference between fibroblasts and hematopoietic cells might also be explained by the different growth characteristics of the cell types studied: Fibroblasts divide much more slowly than hemato-poietic cells and it is possible that hematopoietic DKC T1129P cells require a stronger therapeutic effect than DKC T1129P fibroblasts for an improvement in survival. This difference of the effect of this mutation in either fibroblast or hematopoietic cells might also be reflected by the most severely affected hematopoiesis of the homozygous TERT T1129P patients, whereas the skin and other epithelial tissues were not clinically affected.

Furthermore, the severity of the telomerase mutation may render cells completely unable to re-elongate telomeres. It should also be considered that continuous presence of rapamycin in our experiment might have had additional toxic effects preventing cell division.

In summary, we report a novel hereditary T1129P TERT mutation that leads to DKC with aplastic anemia that can be attributed to a severe reduction and functional impairment of CD34+ hematopoietic stem cells. Functional analyses to find a therapeutic alternative for this serious condition revealed that rapamycin treatment prolonged survival and decreased levels of cellular senescence in treated skin fibroblasts. However, impaired HSC function could not be restored by rapamycin mediated mTOR inhibition, as colony forming capacity in vitro and multilineage engraftment potential in xenotransplanted mice was unchanged in the presence of rapamycin. Our data argue against a therapeutic use of mTOR inhibitors to treat aplastic anemia in DKC patients with TERT mutations.

## MATERIALS AND METHODS

### Ethics statement

Investigation has been conducted in accordance with the ethical standards and according to the Declaration of Helsinki and according to national and international guidelines and has been approved by the authors' institutional review board.

### Genotype analysis

EDTA-blood samples of the patients and their family were obtained after informed consent had been given. DNA was prepared according to standard protocols (QIAampDNA Blood Mini Kit, Qiagen, Germany). The genebank accession number used was NM_198253 for the cDNA and amino acid sequences.

### Telomere length measurement via flow-FISH

Telomere length in lymphocytes and granulocytes from the peripheral blood of our patients was analyzed using flow-FISH as previously described [40, 52, 53]. Briefly, samples were analyzed in triplicates with and without Alexa488-(C3TA2) PNA staining (Panagene, Daejeon, South Korea). Granulocytes, lymphocytes and cow thymocytes were identified based on forward scatter and LDS 751 staining. Cow thymocytes with known telomere length were used as an internal control to calculate telomere length in kilobases. To determine the percentiles, linear regression on 104 blood samples from healthy donors was carried out [54, 55].

### Exome capture and Illumina sequencing

Exome capture was carried out with the SureSelect Target Enrichment Kit v4 (Agilent, Santa Clara, CA) according to manufacturer's instructions (version 1.7, July, 2014). DNA concentration was determined with the Qubit fluorometer using the BR dsDNA Assay (Qubit 2.0, Invitrogen Life Technologies, Grand Island, NY). 3 μg of genomic DNA was sheared using Covaris S2 instrument (Covaris, Woburn, Mass, USA) to a mean size of 150-200bp. 500 ng of the library was subjected to hybridization with the SureSelect baits for 16 hours at 65 °C. Fragments captured in hybridization were indexed, amplified and sequenced in a paired end 100-bp mode using an Illumina HiSeq2000 deep sequencing instrument (v3 sequencing chemistry; Illumina, San Diego, CA).

### Analysis of the whole exome sequencing data

Single nucleotide variant (SNV) calling was performed with the Genome Analysis Toolkit (GATK) and SAMtools mpileup [56, 57]. For GATK, the data were recalibrated with dbSNP v132 and the 1000 Genomes Project Indel release from July 5, 2011 (http://1000genomes.org). Subsequently, SNVs were called by using GATK's Unified Genotyper on the recalibrated data. All GATK calls were annotated for strand bias, low mapping quality, and SNV clusters. The GATK resulting SNV calls were intersected with the SAMtools mpileup SNV calls. All SNVs were intersected with information on gene coding regions by using the Annotation of Genetic Variants framework (ANNOVAR) [58]. By using RefSeq gene annotations, the SNVs were classified as nonsynonymous SNVs affecting protein-coding regions. ANNOVAR was also used to compute the overlap with dbSNP v132 (http://www.ncbi.nlm.nih.gov/projects/SNP), and the October 2011 SNV releases of the 1000 Genomes Project (http://1000genomes.org). Additionally the SNVs were filtered using an in-house database, which is based on more than 25 deeply sequenced (>30x coverage) human genomes and which includes sites that are commonly identified as false-positive SNVs by using GATK and SAMtools mpileup. Following these additional filtering steps, candidate SNVs were evaluated computationally to assess the possible effect of an amino acid substitution on the structure and function of the respective protein by using SIFT and PolyPhen-2 [30, 59].

To verify the results obtained by whole exome sequencing, the coding regions of the TERT gene were PCR-amplified and sequenced (GATC Biotech AG, Germany). Primer sequences used for fragment amplification and sequencing are available on request.

### Plasmid constructs

The 3xHA-TERT in pCDNA-minigene was kindly provided by Steven Artandi over addgene (pCDNA-3xHA-hTERT, addgene plasmid #51637) [60]. Functionality of this TERT construct with 3xHA at the N-terminus has been previously shown by immortalization of human fibroblasts that lack TERT expression [60].

The 3xHA-TERT T1129P minigene was constructed using the following primers:

forward primer Asc1Tert: CGGGGGCGCGCCCCGCGCGCTCCCCG
reverse primer Pac1Tert: AGACTTAATTAATCAGTCCAGGATGGGCTTGAAGTCTG.

After PCR amplification, the PCR products were digested with AscI and PacI and inserted in an AscI and PacI digested pCDNA vector. The identity of all constructs was confirmed by DNA sequencing (GATC Biotech AG, Germany).

The TERC minigene (pBS U3-hTR-500, addgene plasmid #28170) was kindly provided by Kathleen Collins over addgene [61].

### Cell culture and transient transfection

HeLa-cells were grown in Dulbecco's modified Eagle's medium (DMEM) supplemented with 10% FCS and 1% P/S at 37°C and 5% CO_2_. Cells were transiently transfected by calcium phosphate precipitation in 6-well plates using 3μg of the test construct DNA as described before [62].

### Immunocytochemistry

For immunocytochemical detection of the HA-epitope, transfected cells that had been grown on coverslips in 6-well plates were fixed in 4% paraformaldehyde in phosphate-buffered saline (PBS) for 20min at 4°C and pre-treated with 5% FCS in PBS+/−T (0.1% Triton X-100 in PBS) for 20min at RT to block unspecific antibody binding and to permeabilize the cells. The cells were then incubated with a mouse monoclonal anti-HA antibody (Sigma-Aldrich) at 1:1,000 in 3% FCS/PBS. Immunoreactivity was visualized by a goat anti-mouse secondary antibody conjugated to AlexaFluor488 (CellSignaling) (1.1,000 in 3% FCS/PBS). Before mounting the coverslips upside down in moviol 4-88 (Carl Roth) on slides, cells were washed 3x with PBS and nuclei were visualized by staining with DAPI (4′-6-Diamidino-2-phenylindole, 10mg/ml stock solution, Sigma-Aldrich), diluted 1:10,000 in PBS for 10min at RT.

### Microscopy and quantification

Cells were imaged in the 405 and 488nm laser channels (DAPI excitation 405nm, emission 455nm; AlexaFluor excitation 488nm, emission 525nm) using a spinning-disk confocal microscope (Perkin Elmer ERS-6 with a Hamamatsu C9100-50 camera). The system incorporated a Nikon Eclipse TE2000-U inverted microscope using a Nikon 100x objective. Perkin-Elmer Ultraview ERS software and Volocity 6.3 software (Improvision, Lexington, MA) were used for acquisition. Images were subsequently cropped in Adobe Photoshop CS2. Cropped images were imported into Corel Draw X5 for the final figures presented.

For quantification of nuclear accumulation and clustering in the nuclei, 100 cells each from 3 independent transfections have been assessed and counted visually for the presence of nuclear staining and/or nuclear clustering. For statistical analysis a student's unpaired two-sided t-test was used.

### Patient data and material

Patient data and samples were acquired and the patient was treated in accordance with the Helsinki Declaration of 1975.

After written consent, the bone marrow sample was taken according to standard procedures as a necessary routine assessment in bone marrow failure patients to determine cellularity and further cytogenetic aberrations.

Skin fibroblasts were obtained by standard procedures from underarm skin under local anesthesia and grown in DMEM (LifeTechnologies) media supplemented with 10% FCS, 1% P/S and 1% fungizone (LifeTechnologies) at 37°C in 5% CO_2_.

### Determination of population doublings in skin fibroblast cultures

Monolayers were dissociated with trypsin/EDTA and resuspended cells in complete medium. To check for viability, cells were diluted 1:2 with trypan blue (LifeTechnologies). Viable cells remained unstained. Viability and number were determined using a hemacytometer (improved Neubauer). Population doublings were calculated using the following equation: PD=X + log2(Y/I) where: X = initial PD I = cell inoculum (number of cells plated in the flask) Y = final cell yield (number of cells at the end of the growth period).

### Beta-galactosidase (Δ-Gal) Staining + DAPI

Fibroblasts were cultured in DMEM/10% FCS/1%PenStrep containing either DMSO (negative control) or 5 nM rapamycin dissolved in DMSO. For several time points cells were semi-confluently seeded on 18mm-coverslips in DMEM/10%FCS/1%PenStrep at 37°C and 5% CO^2^. Senescence associated Δ-Gal staining was performed according to a modified published protocol [63]. After 24h cells were washed twice with PBS and fixed with 0.1% glutaraldehyde at room temperature for 15 minutes. Following two washing steps with PBS, fibroblasts were stained for Δ-galactosidase using a 0.1%Δ-Gal / 5 mM Potassium hexacyano-ferrate (II)/5 mM Potassium hexacyano-ferrate (III)/2 mM MgCl_2_ / 7.4mM Citric Acid / 150 mM NaCl- solution at pH6 for 14-16 hours at 37°C. Nuclei were visualized by staining with DAPI (4′-6-Diamidino-2-phenylindole, 10mg/ml stock solution, Sigma-Aldrich), diluted 1:10,000 in PBS for 20min at RT. Two additional washings with PBS were performed before embedding coverslips upside down in moviol 4-88 (Carl Roth) on slides. Cells were observed at 20-fold magnification on an Olympus CellR microscope and pictures taken at brightfield and fluorescent light (Ex 330-385, Em LP420), respectively. Depicted overlays of brightfield and fluorescence were merged in Fiji.

### Quantification of Δ-Gal positive cells

The self-written macro Nuclei_PeripheryMeasure was used to count the number of cells with a cytosolic signal above a user-defined threshold within a cell population. As output, the number of criteria-matching cell counts with respect to the total number of cells is provided. The macro works on images or image stacks with at least two channels. In short, the macro takes the nuclear signal (here DAPI) in one channel as reference for individual cells. Segmentation of nuclei is done by intensity thresholding. The corresponding nuclear areas are registered and used to create binary images as masks. In order to measure the cytosolic signal of the second channel (here Δ-Gal), dilations are performed on the binary images in a user-defined manner to match cell dimensions. The resulting mask images with intensity values 0 (background) and 1 (foreground) are multiplied with the images of the second channel to select the areas for measurement. For the analysis, the user can specify both a general signal intensity threshold and a minimal number of pixels required above that threshold for positive counts. The software is available as ImageJ macro and can be downloaded from http://www.zmbh.uniheidelberg.de/Central_Services/Imaging_Facility/2D_ImageJ_Macros.html [64]. The following settings were used to define Δ-Gal positive cells in the programmed plugin: Find and add nuclei to the ROI manger (yes). Substract background (yes), maximum nuclei radius 80 pixels, minimum nuclei size 300 pixels, maximum nuclei size 4000 pixels, minimum circularity 0, maximum circularity 1. Clear ROI manager (yes). Surrounding analysis: surrounding distance (dilation) 5, threshold to detect intensity: 120, amount of pixels over threshold: 100. For quantification an average number of 200 DAPI stained cells were counted.

### FACS analysis, CD34+ magnetic bead enrichment and colony forming unit assays

Bone marrow mononuclear cells were isolated by gradient centrifugation using Histopaque-1077 (Sigma) and labeled with APC-eFluor780-conjugated anti-CD34 (4H11, eBioscience), Alexa-Fluor 700-conjugated anti-CD38 (HIT2, eBioscience), a cocktail of APC-conjugated lineage antibodies consisting of anti-CD4 (RPA-T4), anti-CD8 (RPA-T8), anti-CD11b (ICRF44), anti-CD20 (2H7), anti-CD56 (B159, all BD Biosciences), anti-CD14 (61D3), anti-CD19 (HIB19) and anti-CD235a (HIR2, all eBiocience) and DAPI (Sigma). FACS analysis was performed on LSR Fortessa (BD Biosciences).

CD34+ cells from MNC were isolated using MACS enrichment columns (Miltenyi Biotec) according to the manufacturer's instructions. CD34+ cells were plated in methylcellulose medium (MethoCult H4434; StemCell Technologies) with 5nM rapamycin or DMSO. Colonies were counted after 14 days and pictures were taken with a Nikon Eclipse Ti microscope.

### Mouse transplantation and in-vivo rapamycin treatment

Animals were housed under specific pathogen-free conditions at the central animal facility of the German Cancer Research Center (DKFZ). All animal experiments were approved by the Regierungspräsidium Karlsruhe under “Tierversuchsantrag G210/12”.

Female NSG mice with 8 weeks of age were sub-lethally irradiated (175 cGy) one day before the cells were injected in the femoral bone marrow cavity. For the healthy control 10^5^ CD34+ cells were injected along with 5 x10^5^ MSCs. For the patient 10^6^ CD34-negative MNCs cells were injected along with 5 x10^5^ MSCs. 90 day slow release implantable pellets (Innovative Research of America, USA) with 9mg rapamycin/pellet or placebo were implanted subcutaneously on the day of transplantation.

Recipient mice were analyzed 12 weeks post transplantation. Bone marrow cells were labeled with PE-conjugated anti-human CD45 (2D1), APC-eFluor780-conjugated anti-mouse CD45 (30-F11), PE-Cy5-conjugated anti-human CD3 (UCHT1), APC-labeled anti-human CD19 (HIB19), PE-Cy7-conjugated anti-human CD33 (WM-53, all from ebioscience) to assess multilineage human hematopoietic engraftment.
